# CD133 overexpression correlates with clinicopathological features of gastric cancer patients and its impact on survival: A systematic review and meta-analysis

**DOI:** 10.18632/oncotarget.5714

**Published:** 2015-10-22

**Authors:** Li Yiming, Guo Yunshan, Ma Bo, Zang Yu, Wei Tao, Liang Gengfang, Fan Dexian, Cui Shiqian, Jiang Jianli, Tang Juan, Chen Zhinan

**Affiliations:** ^1^ Cell Engineering Research Centre, State Key Laboratory of Cancer Biology, The Fourth Military Medical University, Xi'an, Shaanxi, China; ^2^ Department of Pharmacology, School of Pharmacy, The Fourth Military Medical University, Xi'an, Shaanxi, China; ^3^ Department of Respiratory Disease, Tangdu Hospital, The Fourth Military Medical University, Xi'an, Shaanxi, China; ^4^ Department of Digestive Surgery, Huawan People Hospital, Zhuyang, Guangxi, China; ^5^ Therapeutic and Preventive Research Center of Digestive System Neoplasm, Zhuyang, Guangxi, China

**Keywords:** CD133, gastric cancer, CSC, IHC

## Abstract

**Background:**

CD133 is one of the most commonly used markers of cancer stem cells (CSCs), which are characterized by their ability for self-renewal and tumorigenicity. However, the clinical and prognostic significance of CD133 in gastric cancer remains controversial. To clarify a precise determinant of the clinical significance of CD133, we conducted a systematic review and meta-analysis to elucidate the correlation of CD133 overexpression with prognosis and clinicopathological features of GC patients.

**Methods:**

A search in the Cochrane Library, Pubmed, Medline, Web of Knowledge and Chinese CNKI, CBM (up to Jun 30, 2015) was performed using the following keywords gastric cancer, CD133, AC133, prominin-1, etc. Electronic searches were supplemented by hand searching reference lists, abstracts and proceedings from meetings. Outcomes included overall survival and various clinicopathological features. Two reviewers independently screened the literature according to the inclusion and exclusion criteria, extracted the data, and assessed the methodological quality of the included studies, and then RevMan 5.2.0 software was used for meta-analysis.

**Results:**

A total of 603 gastric cancer patients from 8 studies were included. The results of the meta-analyses showed that, there were significant differences of CD133 expression in the following comparisons: gastric cancer tissues vs. normal esophageal tissue (OR = 3.49, 95% CI [2.48, 490], *P* < 0.00001), lymph node metastasis vs. non-lymph node metastasis (OR = 2.75, 95% CI [1.99, 3.81], *P* < 0.00001), distant metastasis vs. non-distant metastasis (OR = 2.38, 95%CI [1.47, 3.85], *P* < 0.0004), clinical stages III~IV vs. clinical stages I~II (OR = 2.83, 95% CI [2.13, 3.76], *P* < 0.00001), as well as the accumulative 5-year overall survival rates of CD133-positive vs. CD133-negative patients (OR = 0.23, 95% CI [0.16, 0.33], *P* < 0.00001).

**Conclusion:**

Overexpression of CD133 is associated with lymph node metastasis, distant metastasis, poor TNM stage. Additionally, CD133-positive gastric cancer patients had worse prognosis. Our results indicate that CD133 may be involved in the carcinogenesis of gastric cancer. Evaluation of cytoplasmic CD133 overexpression in gastric cancer tissue sections may be useful in the future as a novel prognostic factor. Nevertheless, due to the poor quality and small sample size of included trials, more well-designed multi-center randomized controlled trials should be performed.

## INTRODUCTION

Gastric cancer ranks fourth (after lung, breast and colorectal) in incidence and second (after lung cancer) in mortality among all cancers worldwide. Nearly one million people are diagnosed with gastric cancer every year worldwide, among which of 70% are in developing countries and more than 50% in East Asia, especially China and Japan [1]. Although its incidence, diagnostic studies and therapeutic options have undergone significant changes in the last decades, the prognosis for gastric cancer patients remains poor, especially in more advanced stages [2]. Since first reported to be responsible for the initiation, progression, metastasis and ultimately recurrence of solid cancers in the early half of the 2000s, cancer stem cells (CSCs) have been an active focus in the field of cancer research [3]. CSCs represent a small subpopulation of cells within a tumor that express cell surface markers including CD44, CD24 and/or CD133 [4]. CD133, also called AC133, prominin-1, was initially described as a specific marker to select human hematopoietic progenitor cells and was recognized as an important marker to identify and isolate CSCs later [5–6]. CD133 is a 120-kDa glycoprotein with five transmembrane 5 and is one of the most important stem cell markers in many solid cancers such as brain tumors [7], colon cancer [8], lung cancer [9], liver cancer [10] and prostate cancer [11]. Many studies have correlated the overexpression of CD133 with either survival, recurrence, metastasis or therapy resistance [12]. However, despite the large number of patients with gastric cancer worldwide, CSCs in gastric cancer have not been definitively reported, especially studies evaluating the correlation between the overexpression and clinical significance of CD133 in gastric cancer systematically. Here, based on current evidences, we performed a systematic review of the literature with a meta-analysis to determine the association between CSCs marker CD133 and the clinicopathological characteristics of gastric cancer and to investigate the roles of CD133 in the prognosis of gastric cancer.

## RESULTS

### Literatures information

**Figure 1:**
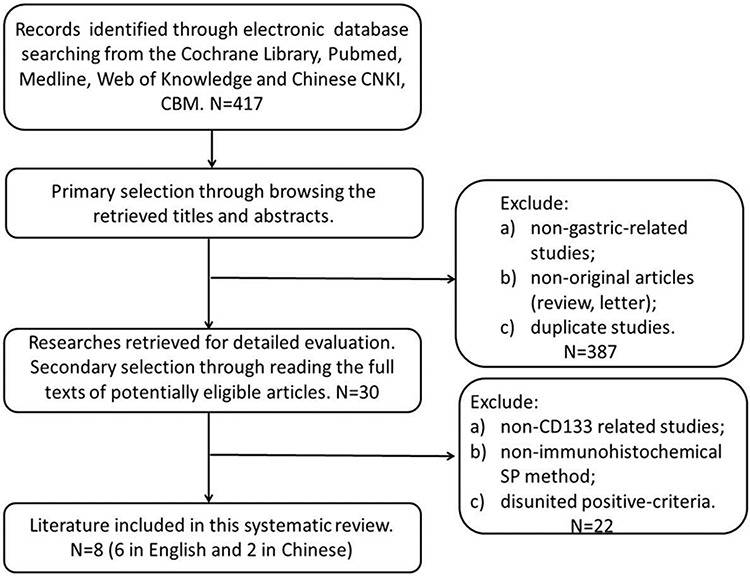
Flow chart for selection of studies

Four hundred and seventeen articles were identified initially using the search strategy above. Through reading titles and abstracts, Three hundred and eighty-seven of those were excluded due to non-gastric-related studies, non-original articles (review, letter) and duplicate studies. After reading full texts, we excluded twenty-two data which couldn't be extracted due to non-CD133 related studies, non-immunohistochemical SP method, disunited positive-criteria. Eventually, there were 8 studies (6 in English and 2 in Chinese) included in the present Meta-analysis [13–20]. Figure 1

### Study characteristics

Based on Asian population, the eight studies included eventually contained 4 from China, 3 from Japan, and the rest 1 from Singapore. A total of 1195 patients were included, containing 607 from China, 476 from Japan, and the rest 112 from Singapore, most of which were male patients (60.4% from 6 studies). The median age ranged from 51.5 to 66 years old (from 5 studies). The total positive rate of CD133 overexpression by IHC is 35.1% from 8 studies (from 9.5% to 65.0%). All detected specimen were derived from gastric cancer tissues by either biopsy or surgical resection, and was proven by IHC on membrane protein level. Furthermore, those studies were divided into four groups according to the following criteria respectively: (1) overexpression of CD133 in gastric cancer tissues and pericarcinoma tissues or normal gastric tissues; (2) overexpression of CD133 in positive and negative lymph node metastasis of gastric cancer tissues; (3) overexpression of CD133 in positive and negative distant metastasis of gastric cancer tissues; (4) overexpression of CD133 in In different clinical stages of gastric cancer tissues; (5) CD133 overexpression and 5-year overall survival. Table 1

**Table 1:** General characteristics of included studies

Studies	Year	Country	Cases (*n*)	Sex (M/F)	Age	Method	CD133 expression rate (%)	Group
Hashimoto	2013	Japan	189	133/56	55~77	IHC	29.6	(2) (3) (4) (5)
Chen	2013	China	152	101/51	23~84	IHC	42.1	(1) (3) (4) (5)
Wakamatsu	2012	Japan	190	—	—	IHC	9.5	(2) (3)
Lu	2012	China	20	—	—	IHC	65.0	(1)
Wang	2011	Singapore	112	76/36	—	IHC	17.0	(2) (3) (4) (5)
Zhang	2011	China	99	69/30	29~83	IHC	29.3	(1) (2) (3)
Ishigami	2010	Japan	97	69/28	40~85	IHC	27.8	(2) (3) (4)
Zhao	2010	China	336	274/62	18~85	IHC	57.4	(3) (5)

### The results of meta- analysis

#### Gastric cancer group vs. control group

**Figure 2:**
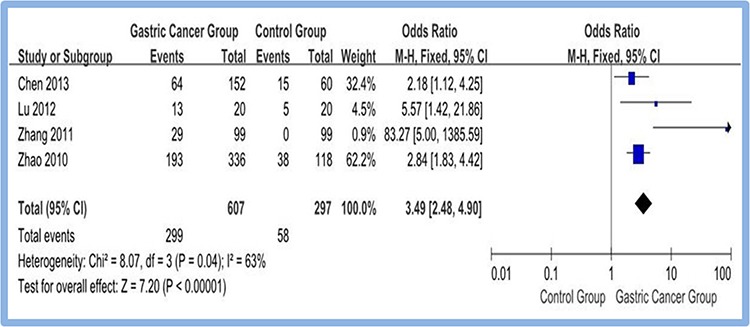
Meta-analysis of overexpression of CD133 in gastric cancer group and control group

A total of four studies [14, 16, 18, 20] reported the overexpression of CD133 in gastric cancer group (gastric cancer tissues) and control group (pericarcinoma tissues or normal gastric tissues). Meta-analysis of random effect model indicated that overexpression rate of CD133 in gastric cancer group is higher than that in control group. The difference between two groups was statistically significant (OR = 3.49, 95% CI [2.48, 4.90], *P* < 0.00001). Figure 2

#### Lymph node (LN) metastasis of gastric cancer tissues: positive group vs. negative group

**Figure 3:**
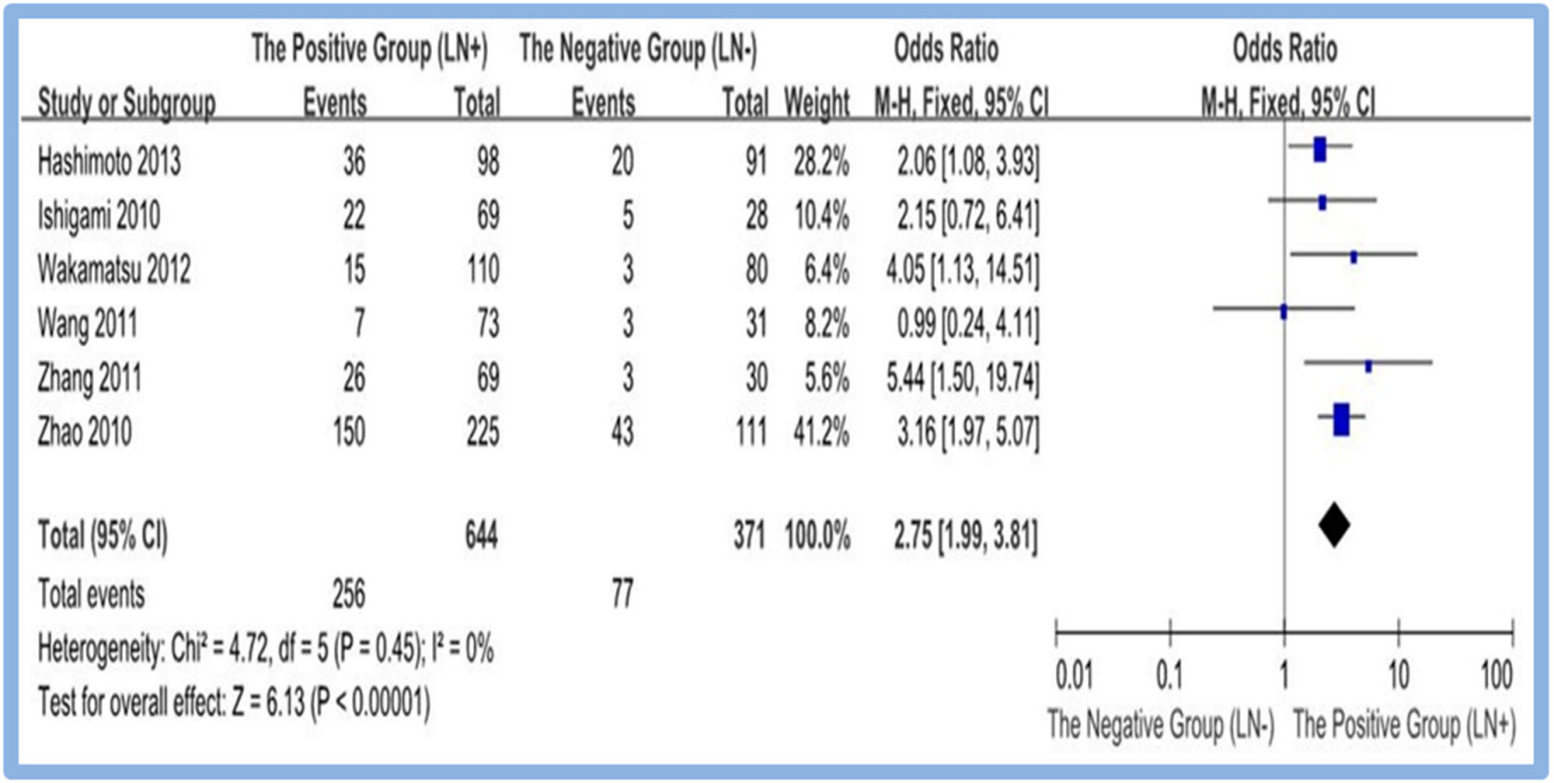
Meta-analysis of overexpression of CD133 in LN(+) and LN(−) gastric cancer group

A scale of six studies [13, 15, 17, 18, 19, 20] reported the overexpression of CD133 in positive and negative lymph node metastasis of gastric cancer tissues. Meta-analysis of random effect model showed that overexpression rate of CD133 in the positive group (LN+) is higher than that in negative group (LN−). The difference between two groups was statistically significant (OR = 2.75, 95% CI [1.99, 3.81], *P* < 0.00001). Figure 3

#### Distant (D) metastasis of gastric cancer tissues: positive group vs. negative group

**Figure 4:**
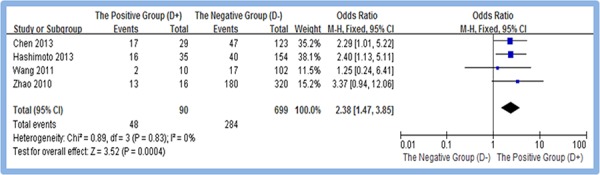
Meta-analysis of overexpression of CD133 in D(+) and D(−) gastric cancer group

A total of four studies [13, 14, 17, 20] reported the overexpression of CD133 in positive and negative distant metastasis of gastric cancer tissues. Meta-analysis of random effect model showed that overexpression rate of CD133 in the positive group (D+) is higher than that in the negative group (D−). The difference between two groups was statistically significant (OR = 2.38, 95% CI [1.47, 3.85], *P* < 0.0004). Figure 4

#### TNM stage of gastric cancer tissues: III~IV stage group vs. I~II stage group

**Figure 5:**
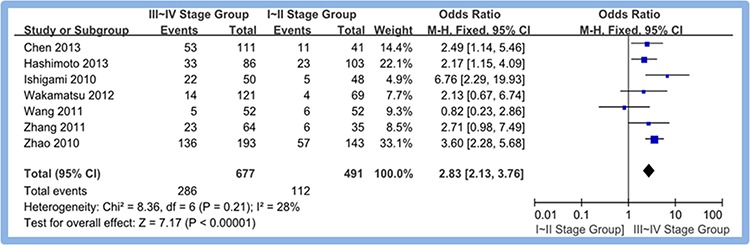
Meta-analysis of overexpression of CD133 in III~IV stage group and I~II stage group

A scale of seven studies [13, 14, 15, 17, 18, 19, 20] reported the overexpression of CD133 in III~IV stage group and I~II stage group of gastric cancer tissues. Meta-analysis of random effect model showed that overexpression rate of CD1133 in the III~IV stage group is higher than that in I~II stage group. The difference between two groups was statistically significant (OR = 2.83, 95% CI [2.13, 3.76], *P* < 0.00001). Figure 5

#### CD133 overexpression and 5-year overall survival

**Figure 6:**
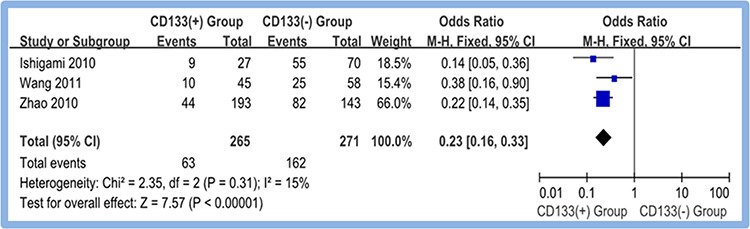
Meta-analysis of 5-year overall survival between CD133(+) and CD133(−) groups

A total of three studies [17, 19, 20] reported the accumulative 5-year overall survival rates of CD133-positive [CD133 (+)] and CD133-negative [CD133 (−)] gastric cancer patients. Meta-analysis of random effect model showed that the CD133-positive group suffered with a significant poor prognosis compared with CD133-negative group. The difference between two groups was statistically significant (OR = 0.23, 95% CI [0.16, 0.33], *P* < 0.00001). Figure 6

### Publication bias

**Figure 7:**
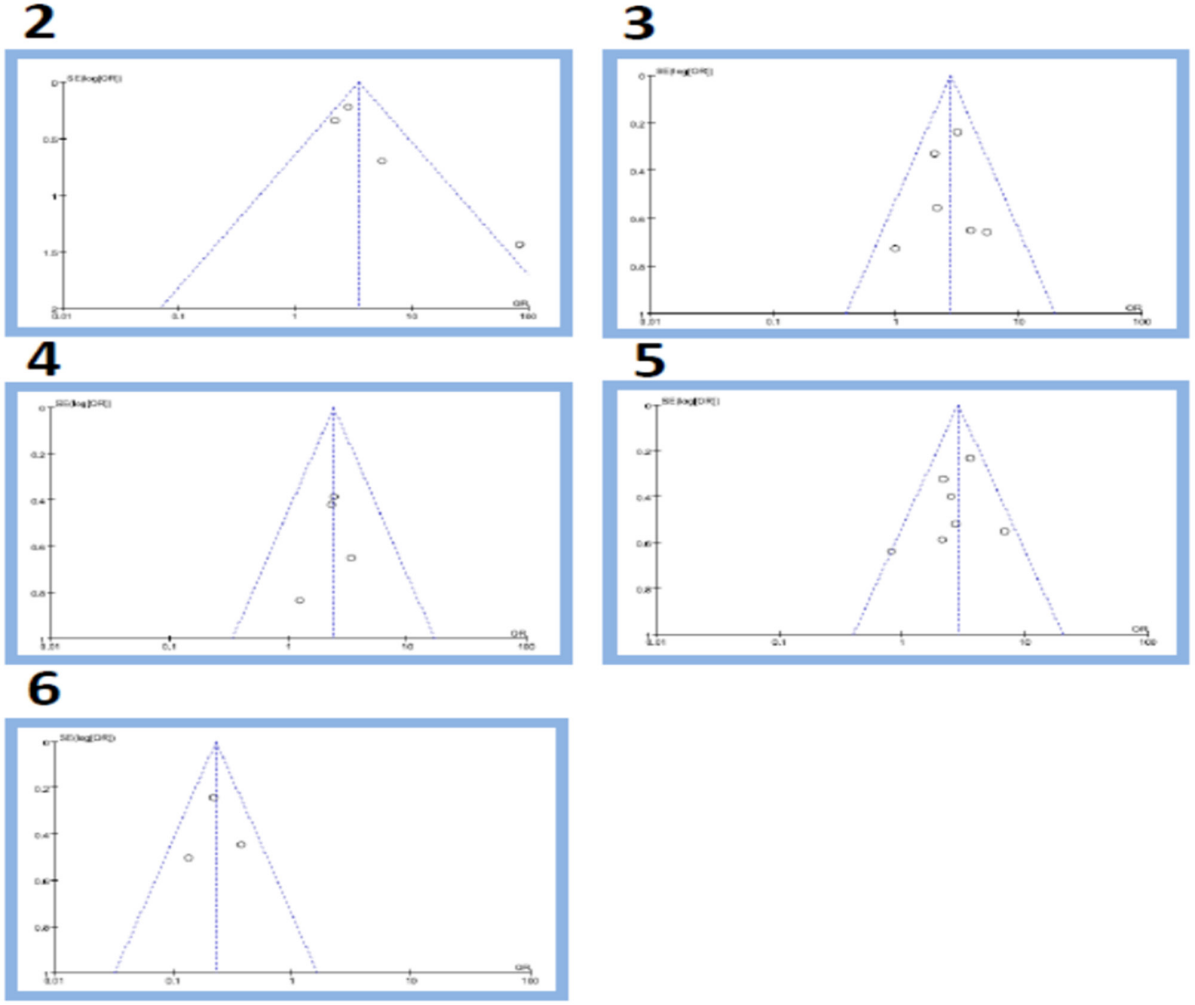
Funnel plot: (2) overexpression of CD133 in gastric cancer group and control group; (3) overexpression of CD133 in LN(+) and LN(−) gastric cancer group; (4) overexpression of CD133 in D(+) and D(−) gastric cancer group; (5) overexpression of CD133 in III~IV stage group and I~II stage group; (6) 5-year overall survival between CD133(+) and CD133(−) groups.

A funnel plot of every two groups in comparison above was applied with RR as the x-axis and SE(RR) as the y-axis, respectively. The plot was symmetric, suggesting that the publication bias was little. Figure 7

## DISCUSSION

Nowadays, CSCs are regarded to be significantly responsible for growth, invasion, metastasis and recurrence of various solid tumors. CSCs are a small subpopulation of cells within tumors with capabilities of self-renewal, differentiation, and tumorigenicity when transplanted into an animal host. Expression of cell surface markers have been used to isolate and enrich CSCs from different tumors such as CD44, CD24, CD29, CD133, aldehyde dehydrogenase1 (ALDH1) and epithelial-specific antigen (ESA) [21]. The discovery of CSCs and their characteristics have contributed to understanding the molecular mechanism of tumor genesis and development, resulting in a new effective strategy for cancer treatment.

Regarding the biological properties of CSCs, many studies indicated that evaluation of CD133 expression in gastric cancer tissue sections may be useful in the future as a novel prognostic factor. Despite a variety of basic and clinical studies on CD133 and gastric cancer, no consensus of opinion has been reached in detail.consensus of opinion consensus of opinionBased on the previous literatures, we systematically reviewed the correlation between overexpression and clinical significance of CD133 in gastric cancer. In the study, we found that overexpression rate of CD133 in gastric cancer group is higher than that in control group by meta-analysis. Moreover, overexpression of CD133 is related to lymph node metastasis, distant metastasis and TNM stage of gastric cancer. Further, CD133 overexpressed gastric cancer patients was of lower 5-year overall survival in comparison with negative ones. In conclusion, overexpression of CD133 and its clinical-pathological features are closely related in gastric cancer. Patients with CD133(+) have stronger drug resistance, higher relapse rate (28.1% vs 65.8%, *P* = 0.002) and lower 5-year survival rate (47.5% vs 74.0%, *P* = 0.037), compared with patients with CD133(−) [22]. CD133 may play a critical role in the pathophysiology, integration and complementation of gastric cancer. Recently, Yu's study found that inhibition of CD133 gene expression reduces the capacities of gastric cancer cells in proliferation, invasion, clonal sphere formation, and chemo-resistance as well as tumor formation in nude mice, which correlates with our study [23]. Zhu found that CD133 may contribute to the resistance of gastric cancer cells to chemotherapy drug through P-gp, Bcl-2 and Bax, involved with PI3K/Akt signal pathway [24]. Fukamachi found that CD133(+) cells specifically expressed Sox17, of which overexpression inhibits the growth of gastric cancer, suggesting that Sox17 may be a key transcription factor controlling CD133 expression [25]. In addition, Wang's studies indicated that two CD133 miRNA binding site variants, rs2240688 and rs3130, may be potential biomarkers for genetic susceptibility to gastric cancer and possible predictors for survival in gastric cancer patients [26]. Nevertheless, the clinically translational potentials warrant further investigation.

Despite a series of success such as obtaining the monoclonal antibody recognizing the glycosylated epitope of CD133 in 1997 [27] and human CD133 independent of glycosylation in 2005 [28], subsequently the first detection of CD133 expression in gastric cancer tissue of 97 cases in 2010 [29], and following basic and clinical studies, there are still some controversies between the overexpression and clinical significance of CD133 in gastric cancer. Researchers around the world are constantly scrambling to understand the biological and molecular mechanisms that lead to tumor formation, subsequent metastasis, even prognosis. Unfortunately, by now the literatures available concerning CD133 have not clarified its biological functions in CSCs. Shmelkov found that during the metastatic transition, CD133(+) tumor cells might give rise to the more aggressive CD133(−) subset, which is also capable of tumor initiation in NOD/SCID mice [30]. Additionally, Marzesco AM even contemplated the hypothesis that it is the fraction of CD133(−) to have greater invasive or similar capacity [31]. As for the combined markers for the identification of CSCs, no positive results have been given. Yong's study indicated that combined expression of CSCs marker CD44/CD24 was not associated with recurrence of gastric carcinoma among 500 patients [32]. Therefore, more prospective work needs to be conducted on the exact mechanisms underlying the hypothesis.

Although this systematic review aimed to provide the best possible estimate of the correlation between the overexpression and clinical significance of CD133 in gastric cancer, it has several limitations. First, the numbers of the studies and patients included in the current meta-analysis are relatively small. Secondly, all of the studies are based on Asian population, including 4 from China, 3 from Japan, and the rest 1 from Singapore. As is known to all, there are significant differences such as etiology, biology features, clinical types, and prognosis in the risk of gastric cancer in different ethnic groups within a given geographical area. Due to lack of statistics on western people, we can not get access to the overexpression rate of CD133 in western patients. In virtue of several limitations and not very steady combined results, further large well-designed prospective cohort studies with better exposure assessment are warranted to confirm the findings from our study and provide a higher level of evidence.

## MATERIALS AND METHODS

### Literature search strategy

A comprehensive literature search of electronic databases the Cochrane Library, Pubmed, Medline, Web of Knowledge and Chinese CNKI, CBM was performed up to Jun 2014. Search strings of PubMed was (((“cd133” [Title/Abstract]) OR “ac133” [Title/Abstract]) OR “prominin 1” [Title/Abstract]) AND (((“stomach neoplasms” [MeSH Terms]) AND “carcinoma” [MeSH Terms] OR “gastric cancer” [Title/Abstract]). The reference lists of relative articles were also screened to further identify potential studies.

### Selection criteria

To be eligible for inclusion in this systematic review, a study was required to meet the following criteria: (1) published in English with the full text available, (2) the use of a case control design or a cohort design, (3) the availability of data to allow the estimation of the hazard ratio (HR) for survival with a 95% CI, (4) diagnosis of gastric cancer was proven by immunohistochemistry (IHC) methods, (5) studies of CD133 overexpression based on primary gastric cancer tissue (via either biopsy or surgical), rather than serum or any other kinds of specimen were included. All studies on the correlation of CD133 overexpression with clinicopathological markers and the association of CD133 overexpression on disease-free and overall survival of gastric cancer were included. When duplicate studies were published, only the most recent or most informative was included in the analysis, to avoid overlap between cohorts.

### Data extraction

Data tables were made to extract all relevant data from texts, tables and figures of each included studies, including author, year, country, patient number, detection method, clinicopathological features, positive rates of CD133 overexpression, as well as the overexpression-related survival. Information was carefully extracted from all the eligible studies independently. Differences in the extraction of data were assessed by a third investigator.

### Statistical analysis

Meta-analysis was supplemented when applicable; otherwise, outcomes were presented in a narrative way. Statistical analysis was performed by Cochrane RevMan 5.2.0 (the Cochrane Collaboration, Copenhagen). Dichotomous data were presented as risk ratio (RR) and continuous variables as mean difference (MD), with 95% confidence intervals (95%CI). Statistical heterogeneity was tested using a Chi-square test with significance being set at *p* < 0.10, the total variation among studies was estimated by I-square. A funnel plot was used for assessing the potential publication bias. Comparisons of dichotomous measures were performed by pooled estimates of odds ratios (OR), as well as their 95% CI. Begg's rank correlation method and Egger's weighted regression method were used to assess publication bias (*P* < 0.05 was considered statistically significant).
